# Evolution of mortality attributable to passive smoking in the 27 Brazilian capitals, 2009-2021

**DOI:** 10.1590/1980-549720260017

**Published:** 2026-04-03

**Authors:** Bibiana Wanderlei Flores, Julia Rey-Brandariz, Paulo César Rodrigues Pinto Corrêa, Sofia Ravara, Agustín Montes Martinez, Mónica Pérez-Ríos

**Affiliations:** I Universidad de Santiago de Compostela, Department of Preventive Medicine and Public Health - Santiago de Compostela, Spain.; II Consortium for Biomedical Research in Epidemiology and Public Health - Madrid, Spain.; III Universidade Federal de Ouro Preto, School of Medicine - Ouro Preto (MG), Brazil.; IV Universidade Federal de Minas Gerais, School of Health Sciences - Belo Horizonte (MG), Brazil.; V Universidade da Beira Interior - Covilhã, Portugal.; VI Local Health Unit of Cova da Beira - Covilhã, Portugal.; VII Universidade NOVA de Lisboa, National School of Public Health, Public Health Research Center - Lisboa, Portugal.; VIII Instituto de Investigaciones Sanitarias de Santiago de Compostela - Santiago de Compostela, Spain.

**Keywords:** Tobacco use disorder, Mortality, Brazil, Cardiovascular diseases, Epidemiology

## Abstract

**Objective:**

To estimate the mortality attributable to passive smoking in the population aged 35 years old and older, by gender, in the 27 Brazilian state capitals, from 2009 to 2021.

**Methods:**

A prevalence-dependent method was used, based on the calculation of population attributable fractions (PAF). Deaths from diseases causally related to passive smoking were obtained from the Mortality Information System of the Brazilian Unified Health System (SIM/SUS); prevalence data were taken on SHS exposure were obtained from Vigitel surveys (2009-2021); and relative risks were obtained from a meta-analysis. Mortality attributable to passive smoking and mortality rates were estimated by capital city, year, gender, and cause of death. Trends in crude mortality rates attributable to passive smoking were analyzed using joinpoint regression models.

**Results:**

Passive smoking accounted for 64,913 deaths in all Brazilian state capitals between 2009 and 2021. Cardiovascular diseases were the main cause of death in both genders. The mortality rate attributed to passive smoking decreased from 33.1/100,000 deaths in 2009 to 15.4/100,000 deaths in 2021. This reduction was observed in all 27 Brazilian state capitals, both overall and by gender.

**Conclusion:**

Passive smoking was responsible for 1.4% of all deaths in Brazil during the period 2009-2021 and showed a favorable trend, with rates decreasing by half during the period.

## INTRODUCTION

Passive smoking constitutes a major global public health problem^1^, being responsible for approximately 1.3 million deaths annually worldwide^2^. More than 600 scientific studies have demonstrated its harmful effects^3^, establishing a causal association with lung cancer and ischemic heart disease in adults^4^. In addition, a recent meta-analysis expanded the evidence of causality to other diseases, including chronic obstructive pulmonary disease (COPD), cerebrovascular disease, type 2 diabetes mellitus, asthma, lower respiratory tract infections, and breast cancer^5^.

The impacts of secondhand smoke on health have driven the formulation of effective public policies and population-based strategies, consolidated in the World Health Organization Framework Convention on Tobacco Control (WHO/FCTC) and the WHO’s 2008 MPOWER package^2^. In Brazil, although the WHO/FCTC was ratified in 2005, measures to protect against secondhand smoke began with Law No. 9.294/1996^6^ and its subsequent amendments in 2011 (Law No. 12.546/2011)^7^ and 2014 (Decree No. 8.262/2014)^8^. These amendments included a national ban on the advertising, promotion, and sponsorship of tobacco products; the strengthening of smoking cessation programs through the provision of treatment in primary care within the Brazilian Unified Health System (*Sistema Único de Saúde* - SUS); and the prohibition of tobacco consumption in collective environments, both public and private^7^ ^,^ ^8^. To assess the impact of these measures, it is essential to estimate the prevalence and attributable mortality (AM) of active and passive smoking, as well as to examine potential changes over time.

The first estimate of the prevalence of passive smoking in Brazil was conducted in 1989 through the National Health and Nutrition Survey, which identified a prevalence of 31.7% among adults^9^. In 2003, the Household Survey on Risk Behaviors and Self-Reported Morbidity of Non-Communicable Diseases and Conditions was conducted. According to data from this survey, across the 27 Brazilian capitals, the prevalence of passive smoking ranged from 13% in Campo Grande to 26% in Recife^10^. Since 2009, the Surveillance System for Risk and Protective Factors for Chronic Diseases by Telephone Survey (*Sistema de Vigilância de Fatores de Risco e Proteção para Doenças Crônicas por Inquérito Telefônico -* Vigitel) has annually reported the prevalence of passive smoking in Brazilian capitals. Between 2009 and 2023, a significant reduction in prevalence was observed among adults aged 18 years old or older, decreasing from 12.7% to 6.4% at home and from 12.1% to 7% in the workplace^11^ ^,^ ^12^.

In Brazil, eight studies have estimated mortality attributable to passive smoking ^13^ ^,^ ^14^ ^,^ ^15^ ^,^ ^16^ ^,^ ^17^ ^,^ ^18^ ^,^ ^19^ ^,^ ^20^; however, none analyzed this mortality by federative unit or capital, nor did they include diseases more recently associated with this exposure^5^. Brazil is a country of continental dimensions and exhibits substantial demographic, socioeconomic, and epidemiological inequalities. Therefore, detailed regional estimates are essential to assess the impact of smoke-free environment policies, strengthen evidence of their effectiveness, and inform adaptations according to local needs, particularly among more vulnerable population groups.

Thus, the objective of this study was to estimate the risk attributable to passive smoking in the 27 Brazilian capitals, among the population aged 35 years old or older, by gender, from 2009 to 2021.

## METHODS

### Study design and context

This is a study of mortality attributable to passive smoking in the 27 Brazilian capitals from 2009-2021. Brazil is composed of five major regions: North, Northeast, Central-West, Southeast, and South. These regions are divided into 27 federative units, each with a capital. In 2024, the Brazilian population was estimated at 212,583,750, of which 23.1% (49,175,449) resided in the capitals^21^.

### Statistical methods

To estimate AM to passive smoking, a prevalence-based method was applied, based on the calculation of Population Attributable Fractions (PAF)^22^. The estimates were conducted in accordance with the recommendations of the STREAMS-p guideline^23^.

PAF was calculated using the following formula:

$$PAF=\frac{\left(q+pxRR\right)-1}{q+pxRR}$$

In this formula, PAF represents the population attributable fraction; p denotes the prevalence of passive smoking; q=1-p; and RR represents the risk of death among individuals exposed to passive smoking compared with those not exposed.

AM was obtained by multiplying the PAF by the Observed Mortality (OM):

AM=PAFxOM

### Data sources and study population

Mortality data for the study population were obtained from the death registry database of the Mortality Information System of the Brazilian Unified Health System (*Sistema de Informação de Mortalidade do Sistema Único de Saúde do Brasil* - SIM/SUS). Data for each year were extracted by gender and classified, according to the 10^th^ revision of the International Classification of Diseases (ICD-10), by capital city for the following causes: lung cancer, ischemic heart disease, cerebrovascular disease, COPD, type 2 diabetes mellitus, lower respiratory tract infections, asthma, and breast cancer^5^. For presentation of the results, these causes were grouped into cancer, cardiovascular diseases (CVD), and respiratory diseases.

The prevalence of passive smoking in the study population was calculated by gender and for each of the 27 Brazilian capitals, based on microdata from Vigitel collected between 2009 and 2021. Vigitel is a population-based cross-sectional survey that assesses the adult population (>18 years) residing in Brazilian capitals. The sample size for estimating the prevalence of any factor is determined assuming a 95% confidence interval and a 2% sampling error, resulting in a minimum of 2,000 interviews per capital. Sampling is conducted in two stages: random selection of households with a landline telephone and random selection of one adult resident to respond to the survey. For passive smoking, exposure is assessed using the following questions: “Does anyone living with you usually smoke inside the house?” and “Does any of your coworkers usually smoke in the same environment where you work?” Response options are “yes” or “no.” An individual was considered exposed if a “yes” response was provided to either question.

The risks of death associated with passive smoking were obtained from the study by Flor et al.^5^: ischemic heart disease [1.26 (1.20-1.32)], lung cancer [1.37 (1.30-1.45)], cerebrovascular disease [1.16 (1.11-1.22)], COPD [1.44 (1.21-1.71)], type 2 diabetes mellitus [1.16 (1.09-1.24)], lower respiratory tract infections [1.34 (1.23-1.45)], asthma [1.21 (1.16-1.26)], and breast cancer [1.22 (1.13-1.31)].

### Statistical analysis

AM and respective crude rates of passive smoking were estimated for each Brazilian capital and for each year of the study period (2009-2021), according to gender and cause of death. Ninety-five percent confidence intervals (95%CI) were calculated for the crude rates using the following formula^24^:

(100,000/p) (AM±(1.96x√AM)

Where p represents the denominator of the crude rate, that is, the population used for its calculation; and AM corresponds to the numerator, that is, the number of attributable deaths considered in the calculation of the crude rate.

The trend in gross AM rates was analyzed using joinpoint regression models. Initially, a maximum of three cutoff points was defined based on the Bayesian Information Criterion (BIC)^25^ and a significance level of 5%. The annual percentage change (APC) was calculated with 95% confidence intervals (95%CI). AM estimation was performed using Stata version 17^26^, and the joinpoint regression models were fitted using the Joinpoint Regression Program version 5.3^27^.

### Ethical aspects

This study used secondary data that are publicly available. Resolution No. 466/2012^28^, which addresses research involving human beings, establishes that the use of publicly available data that do not involve the identification or possibility of identification of participants does not require approval from a Research Ethics Committee (REC).

## Declaration of data availability

The database and analysis codes used in the study are available at https://svs.aids.gov.br/download/Vigitel/

## RESULTS

Between 2009 and 2021, passive smoking was responsible for 64,913 deaths among individuals aged 35 years old or older in Brazilian capitals, corresponding to 4.8% of the mortality observed from the causes under study and 1.4% of the mortality from all causes during the same period. Of the total deaths attributable to passive smoking, 59.8% (38,845) occurred in men, with 56.1% (36,396) attributed to CVD. Ischemic heart disease was the leading specific cause of mortality among men (12,817 deaths; 33.6% of total attributable mortality), whereas among women, the leading cause was lower respiratory tract infections (6,628 deaths; 25.4% of total AM) (Supplementary Tables 1 and 2).

**Figure 1.**
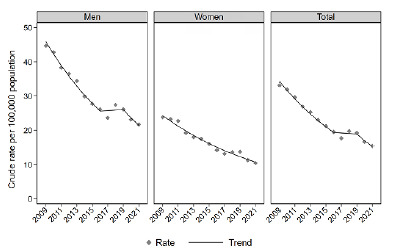
Crude attributable mortality to passive smoking and their trend, for all causes, overall and by gender, in the 27 Brazilian capitals, 2009-2021.

Crude AM from passive smoking decreased in all Brazilian capitals during the study period, declining from 33.1 deaths per 100,000 inhabitants in 2009 to 15.4 in 2021. This reduction was observed in both genders. Among men, rates decreased from 27.7 deaths per 100,000 inhabitants in 2009 to 12.8 in 2021, while among women, rates declined from 13.4 to 5.2 deaths per 100,000 inhabitants over the same period (Figure 1). Overall, this decrease was more pronounced between 2009 and 2016 (APC -7.8; 95%CI -10.6 to -5). In men, three periods were identified: the first showed a decreasing trend until 2016 (APC -8; 95%CI -11 to -4.2); the second showed a stable trend until 2019 (APC 0.5; 95%CI -9.6 to 3.3); and the third showed a decreasing trend between 2019 and 2021 (APC -9.2; 95%CI -14.2 to -2.4). In women, since 2009, the trend in AM rates consistently decreased (APC -6.6; 95%CI -7.5 to -5.7). The APC values and 95%CIs are presented in Table 1.

**Table 1.** Annual Percentage Change and 95% confidence interval by gender in the 27 Brazilian capitals, 2009-2021.

Capital	APC (95%CI)		
	2009-2021		
	Overall	Men	Women
Aracaju	-9.7 (-11.8 to -7.5)	-9.7 (-13.2 to -6)	-9.6 (-12.4 to -6.8)
Belém	-4.6 (-6.8 to -2.3)	-5 (-8.2 to -1.5)	-3.8 (-9.4 to 1.9)
Belo Horizonte	-6.3 (-9.2 to -3.2)	-5.3 (-7.7 to -2.8)	-7.9 (-10.7 to -5)
Boa Vista	-5 (-7.1 to -2.8)	-4.1 (-6.2 to -1.9)	-7.2 (-10.6 to -3.7)
Brasília	-6.1 (-7.7 to -4.4)	-6.2 (-8.6 to -3.6)	-6.3 (-9.3 to -3.3)
Campo Grande	-3.2 (-4.9 to -1.3)	-2.6 (-5.3 to 0.2)	-4.2 (-6.4 to -1.9)
Cuiabá	-8.4 (-12.2 to -4.3)	-7.1 (-10.8 to -3.3)	-10.6 (-14.8 to -6.2)
Curitiba	-8.8 (-11.8 to -5.7)	-8.8 (-11.2 to -6.2)	-8.9 (-12.8 to -4.7)
Florianópolis	-5.5 (-8.2 to -2.7)	-5 (-9.4 to -0.4)	-6.6 (-9.5 to -3.6)
Fortaleza	-4.7 (-7 to -2.3)	-3.9 (-6.3 to -1.5)	-5.7 (-7.2 to -4.1)
Goiânia	-9.0 (-10.6 to -7.4)	-8.3 (-10.8 to -5.7)	-10.1 (-11.4 to -8.7)
João Pessoa	-6.9 (-9.6 to -4.2)	-6.7 (-8.1 to -4.3)	-8 (-11.6 to -4.5)
Macapá	-2.1 (-5.1 to 0.9)	-2.5 (-5.7 to 0.7)	-1.4 (-3.1 to 0.2)
Maceió	-7.2 (-10.6 to -3.8)	-6.1 (-10.6 to -1.4)	-8.9 (-12.8 to -4.7)
Manaus	-3.8 (-5.9 to -1.7)	-2.9 (-6.7 to 0.9)	-5.6 (-8.2 to -2.9)
Natal	-5.8 (-8.3 to -3.3)	-6.2 (-8.8 to -3.5)	-5.3 (-7.3 to -3.3)
Palmas	-3.9 (-7.7 to 0.1)	-3.0 (-9 to 3.3)	-5.8 (-9.2 to -2.2)
Porto Alegre	-6.8 (-8.6 to -5)	-6.1 (-8.9 to -3.3)	-7.6 (-9.8 to -5.4)
Porto Velho	-8.9 (de -10.9 to -6.7)	-9.8 (-12 to -7.6)	-7.5 (-11.4 to -3.3)
Recife	-6.4 (-9.5 to -3.1)	-5.8 (-8 to -3.4)	-7.1 (-9.9 to -4.2)
Rio Branco	-5 (de -9 to -0.9)	-4.7 (-7.5 to -1.7)	-4.7 (-7.5 to -1.7)
Rio de Janeiro	-5 (-8.1 to -1.8)	-5.5 (-9.7 to -1.1)	-4.5 (-7.7 to -1.3)
Salvador	-5.1 (-6.6 to -3.5)	-4.7 (-6.4 to -3)	-5.5 (-8.6 to -2.3)
São Luís	-4.6 (-5.8 to -3.3)	-3.7 (-5.4 to -1.9)	-5.5 (-7.4 to -3.6)
São Paulo	-6.4 (-8 to -4.9)	-5.9 (-7.9 to -3.9)	-7.6 (-9.8 to -5.2)
Teresina	-7 (-8.9 to -5)	-6.9 (-9.4 to -4.4)	-6.9 (-9.7 to -3.9)
Vitória	-4.3 (-7.4 to -1)	-2.9 (-7.3 to 1.6)	-6.4 (-10 to -2.6)

APC: Annual Percentage Change; 95%CI: 95% confidence interval.

Table 2 presents the crude AM of secondhand smoke exposure (per 100,000 inhabitants) in Brazilian capitals in 2009 and 2021. Recife, Pernambuco (PE), registered the largest decrease, declining from 48.4 in 2009 to 17.7 in 2021. The smallest decrease was observed in Palmas, Tocantins (TO), where rates decreased from 26.8 to 22.6 over the same period. Among men, the AM of secondhand smoke exposure decreased in 26 capitals, with the exception of Palmas, where it increased from 34.2 in 2009 to 39.9 in 2021. Among women, a decrease in rates was observed in all capitals.

**Table 2.** Crude attributable mortality to passive smoking (per 100,000 inhabitants) and 95% confidence intervals, by gender, in the 27 Brazilian capitals, 2009-2021.

Capital	Crude attributable mortality rate (95%CI)					
	2009			2021		
	Overall	Men	Women	Overall	Men	Women
Aracaju	33.9 (26.2 to 41.6)	39.6 (27 to 52.2)	29.5 (19.9 to 39.1)	12 (8.2 to 15.7)	18.6 (11.4 to 25.8)	7 (3.2 to 10.8)
Belém	33.6 (28.7 to 38.5)	47.8 (39.1 to 56.6)	22 (16.7 to 27.4)	19.7 (16.5 to 22.9)	28.9 (23.1 to 34.8)	12.4 (9 to 15.8)
Belo Horizonte	22.6 (19.8 to 22.5)	29.1 (24.2 to 33.9)	17.5 (14.2 to 20.9)	13.8 (11.8 to 15.7)	22.2 (18.5 to 26)	7.1 (5.3 to 9)
Boa Vista	17.9 (8.8 to 27.1)	24.5 (9.2 to 39.7)	11.5 (1.1 to 21.9)	11.1 (6 to 16.1)	15.6 (7.1 to 24)	6.6 (1.1 to 12.1)
Brasília	18.3 (15.6 to 21.1)	22.9 (18.4 to 27.4)	14.5 (11.2 to 17.8)	8.1 (6.6 to 9.5)	8.8 (6.6 to 11)	7.5 (5.6 to 9.4)
Campo Grande	34.1 (27.6 to 40.5)	45.8 (34.9 to 56.8)	23.9 (16.6 to 31.3)	25.4 (20.7 to 30.1)	38.5 (30 to 47.1)	14.5 (9.7 to 19.3)
Cuiabá	33.4 (25.6 to 41.2)	39 (26.7 to 51.3)	28.3 (18.4 to 38.2)	7 (4 to 10)	10.6 (5.2 to 15.9)	3.9 (0.8 to 7)
Curitiba	27.5 (23.8 to 31.2)	38.8 (32.2 to 45.3)	18.2 (14.2 to 22.3)	11.2 (9.2 to 13.3)	14.1 (10.6 to 17.5)	8.9 (6.4 to 11.4)
Florianópolis	31.1 (23.1 to 39.1)	42.5 (28.6 to 56.3)	21.5 (12.5 to 30.6)	13.6 (9.3 to 17.9)	19.5 (12 to 27.1)	8.5 (3.9 to 13.2)
Fortaleza	27.9 (24.6 to 31.3)	35.4 (29.7 to 41.1)	22.1 (18.1 to 26)	15.5 (13.4 to 17.7)	21.4 (17.6 to 25.3)	11 (8.6 to 13.4)
Goiânia	41.3 (35.8 to 46.8)	60.6 (50.7 to 70.5)	25.3 (19.4 to 31.1)	12.5 (10 to 15)	18.6 (14.1 to 23.1)	7.4 (4.8 to 10)
João Pessoa	36.8 (29.8 to 43.8)	48.2 (36.1 to 60.3)	28 (19.9 to 36.1)	13.2 (9.6 to 16.7)	20.4 (13.7 to 27.1)	7.7 (4.1 to 11.3)
Macapá	18.3 (10.3 to 26.3)	25.4 (11.9 to 38.9)	11.6 (2.7 to 20.4)	13.4 (8.2 to 18.6)	16 (7.8 to 24.3)	10.9 (4.5 to 17.4)
Maceió	35 (28.8 to 41.2)	44.4 (33.9 to 54.9)	27.7 (20.4 to 35)	13.5 (10.2 to 16.8)	18.7 (12.8 to 24.7)	9.6 (5.9 to 13.3)
Manaus	24.1 (20.1 to 28.2)	32.7 (25.9 to 39.6)	16.3 (11.7 to 20.9)	14.7 (12.2 to 17.2)	20.7 (16.3 to 25)	9.4 (6.7 to 12.2)
Natal	52.2 (44.3 to 60)	71.3 (57.4 to 85.2)	37.3 (28.5 to 46.2)	22.8 (18.4 to 27.3)	30.1 (22.4 to 37.8)	17.1 (12 to 22.2)
Palmas	26.8 (14.2 to 39.5)	34.2 (14.2 to 54.2)	19.3 (4.1 to 34.5)	22.6 (14 to 31.2)	39.9 (23.2 to 56.6)	7.7 (0.8 to 14.5)
Porto Alegre	40.2 (35.4 to 45)	52.6 (44.3 to 61)	30.9 (25.3 to 36.4)	15.3 (12.6 to 18)	21.4 (16.6 to 26.2)	10.6 (7.6 to 13.6)
Porto Velho	25.4 (17 to 33.8)	37.8 (23.4 to 52.2)	12.8 (4.4 to 21.3)	8.3 (4.5 to 12)	11.3 (5.2 to 17.4)	5 (0.9 to 9.2)
Recife	48.4 (43.2 to 53.7)	61.3 (52.2 to 70.4)	38.9 (32.7 to 45.1)	17.7 (14.9 to 20.6)	26.1 (20.9 to 31.4)	11.6 (8.6 to 14.6)
Rio Branco	20.1 (11.4 to 28.7)	28.4 (13.5 to 43.4)	12.5 (3.1 to 21.9)	13.9 (8.2 to 19.6)	19.2 (9.3 to 29.1)	9.3 (2.8 to 15.7)
Rio de Janeiro	38.9 (36.7 to 41.4)	56.6 (52.5 to 60.7)	25.1 (22.8 to 27.5)	21.8 (20.3 to 23.3)	31.6 (28.9 to 34.4)	14.1 (12.4 to 15.7)
Salvador	21.1 (18.4 to 23.9)	26.8 (22.2 to 31.5)	16.7 (13.4 to 19.9)	11.3 (9.6 to 13)	16.8 (13.7 to 20)	7.1 (5.3 to 8.9)
São Luís	32.9 (26.9 to 38.9)	47.4 (36.4 to 58.2)	21.3 (14.8 to 27.7)	18.4 (14.7 to 22.2)	24.7 (18.1 to 31.3)	13.7 (9.5 to 18)
São Paulo	34.7 (33 to 36.3)	46.2 (43.4 to 49.1)	25.3 (23.5 to 27.2)	14.5 (13.6 to 15.4)	19.5 (17.9 to 21.1)	10.5 (9.4 to 11.5)
Teresina	48 (40.1 to 55.9)	72.8 (58.2 to 87.5)	28.6 (20.5 to 36.7)	21.9 (17.3 to 26.5)	31.2 (22.8 to 39.7)	15.1 (10.1 to 20.1)
Vitória	38.9 (28.6 to 49.1)	46.6 (29.7 to 63.4)	32.7 (20.1 to 45.3)	19.7 (13.5 to 26)	31 (19.3 to 42.7)	10.7 (4.6 to 16.9)

95%CI: 95% confidence interval.

**Figure 2.**
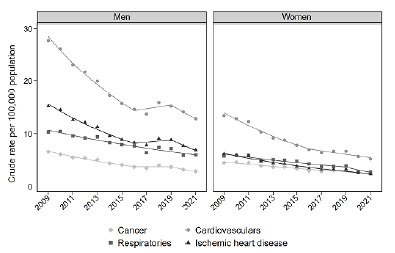
Crude attributable mortality to passive smoking, by cause and by gender, in the 27 Brazilian capitals, 2009-2021.

**Figure 3.**
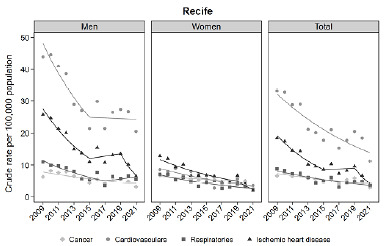
Crude attributable mortality to passive smoking, by cause, overall and by gender, in Recife (PE), 2009-2021.

Analysis by major groups of causes of death showed that CVD presented the highest rates throughout the study period in all Brazilian capitals (Figure 2). Recife/PE was the capital with the highest AM from passive smoking due to CVD over the entire period (Figure 3). It is noteworthy that in Belém, Pará (PA), CVD were the leading cause of death until 2020; and in Rio Branco, Acre (AC), until 2021, when they were surpassed by respiratory diseases (Supplementary Figures S1 and S2).

## DISCUSSION

Between 2009 and 2021, passive smoking was responsible for 64,913 deaths in the 27 Brazilian state capitals; 6 out of 10 occurred in men, mainly attributed to CVD. The leading specific cause of death related to passive smoking was ischemic heart disease among men and lower respiratory tract infections among women. Over the study period, AM from passive smoking decreased substantially, from 6,290 deaths in 2009 to 3,931 deaths in 2021.

The decrease in exposure to secondhand smoke occurred in all capitals, although with varying magnitudes, possibly related to socioeconomic factors. Recife (PE) showed the greatest reduction in both genders. A previous study conducted in Brazil also identified Pernambuco as the federative unit with the greatest decrease in tobacco exposure rates between 1990 and 2017^29^. Palmas (TO), Macapá (Amapá - AP), Rio Branco (AC), and Boa Vista (Roraima - RR), showed the smallest reductions in AM. These capitals have a Human Development Index (HDI) below the national average^30^. HDI is an indicator that assesses the level of development of a population based on three fundamental dimensions: education, income, and longevity^31^. A study conducted in Brazil in 2011 reported a higher prevalence of secondhand smoke exposure at home and at work among individuals with lower income and lower levels of education^32^, highlighting the importance of addressing inequalities in the fight against tobacco consumption and strengthening existing policies and programs^33^.

In 2021, Campo Grande, Mato Grosso do Sul (MS), and Natal, Rio Grande do Norte (RN), presented the highest mortality rates from passive smoking. Campo Grande stands out for having the highest prevalence of tobacco use and for being among the ten capitals with the highest prevalence of passive smoking at home^11^. Natal, located in a federative unit with an HDI below the national average, faces limitations in access to health services, which may compromise the effectiveness of tobacco control policies and contribute to higher mortality rates^30^. In contrast, Cuiabá, Mato Grosso (MT), and Brasília, Federal District (DF), presented the lowest AM. Both capitals have a high HDI, a condition associated with better implementation of tobacco control policies and lower prevalence of tobacco consumption and passive smoking^30^. In Cuiabá (MS), the prevalence of passive smoking at home is among the lowest in the country^11^.

Three previous studies that estimated AM of passive smoking in Brazil using the Global Burden of Disease (GBD) methodology^18^ ^,^ ^19^ ^,^ ^20^ reported AM rates higher than those observed in the present study: 44.5/100,000 inhabitants^18^ and 32.8/100,000 inhabitants^19^, compared with 19.2/100,000 inhabitants in this study. Another recent study^20^ presented results stratified by gender: 14.2/100,000 inhabitants among men and 6/100,000 inhabitants among women, compared with 3.7/100,000 inhabitants among men and 1.5/100,000 inhabitants among women in the present study. Although the methodology used here is similar to that applied in the GBD studies, there are differences in the calculation procedures, included causes, age groups, and geographic scope. The GBD methodology adjusts for uncoded deaths and redistributes the so-called garbage codes (GC), which was not performed in the present study due to the low proportion of these codes in Brazil (<5%).

Malta et al. analyzed the effect of GC redistribution on trends in mortality from chronic diseases in Brazil (2010-2019), comparing raw data from SIM, data corrected for GC and underreporting, and data from the GBD. Despite little change in temporal trends, the corrected rates were significantly higher, with heterogeneous variation among the federative units^34^. Regarding causes of death, 8 were included in the present study: ischemic heart disease, CVD, lung cancer, breast cancer, lower respiratory tract infections, type 2 diabetes mellitus, COPD, and asthma. Reitsma et al.^19^ excluded asthma; São José et al.^18^ analyzed only cancer; and Salerno et al.^20^ included only lung cancer. The age range of the population also differed: 35 years old or older in the present study; 40 years old or older in the study by São José et al.^18^; 25 years old or older in the study by Reitsma et al.^19^; and 20 years old or older in the study by Salerno et al.^20^. The present study also differs from that of São José et al.^18^, which considered the combined burden of active and passive smoking. Furthermore, it is important to note that the present study is limited to the population of Brazilian state capitals, representing approximately 23% of the total population of the country, whereas the other studies covered the national population.

According to previous studies conducted in Brazil^15^ ^,^ ^16^, the AM associated with passive smoking is higher among men. This difference may be explained by the higher prevalence of exposure to secondhand smoke in the workplace among men (10.2%), which is more than double that observed among women (4.3%)^11^. It is important to highlight that differences in the AM of passive smoking - both overall and by specific cause and gender (ischemic heart disease among men and respiratory diseases among women) - were also reported in another study conducted by the same research group on the risk of tobacco use in Brazil^35^.

AM from secondhand smoke in South America are scarce^36^. A study conducted in Chile estimated 70 lung cancer deaths attributable to secondhand smoke^37^. Using the same methodology, the author estimated 387 deaths in Brazil^15^. A recent study reported disparities in AM for tracheal, bronchial, and lung cancer: Uruguay had the highest rates among men (42.6/100,000 inhabitants), and Argentina among women (8.8/100,000 inhabitants). Peru presented the lowest rates in both genders (3.6 and 0.7/100,000 inhabitants). Brazil occupied an intermediate position, ranking sixth lowest among men (14.2/100,000 inhabitants) and third lowest among women (6/100,000 inhabitants)^20^.

This study has limitations related to the estimation methods and data sources used. In estimating AM, the latency period between exposure to passive smoking and death was not considered, as both indicators were analyzed concurrently in time. This is due to the lack of specific information on latency periods, which likely vary according to the cause and level of exposure. Regarding prevalence, Vigitel is based on self-reported information, which may lead to underestimation of the prevalence of passive smoking due to recall bias. There is also the possibility of selection bias, since the sample is drawn from a telephone directory, excluding individuals without access to this service, although post-stratification weights are applied to minimize this effect. With respect to observed mortality, GC (approximately 5% of deaths) were not redistributed, as this procedure would introduce additional uncertainty into the AM estimates. As a result, mortality may be underestimated, although it is assumed that this underestimation is of small magnitude.

It is important to consider that the COVID-19 pandemic may have influenced both the quality of mortality records and the coding of causes of death in 2020 and 2021. Excess mortality and the initial absence of a specific code for COVID-19 may have led to misclassification of causes of death. Due to the lack of national studies estimating specific relative risks (RR) for the association between passive smoking and mortality in Brazil, RR estimates from a recent meta-analysis were used^5^. In addition, although potential variations in the age distribution of populations residing in Brazilian capitals over the 13-year period are acknowledged, mortality rates were not age-adjusted, as the objective of this study was to assess the overall burden of passive smoking, without accounting for changes in population age structure.

This study also presents important strengths. For the first time, AM from passive smoking was estimated using more recent causes of death with gender-specific prevalence data, in addition to applying the same calculation procedures and data sources across all Brazilian capitals, allowing direct comparability of results. Moreover, the temporal evolution of mortality AM from passive smoking in Brazilian capitals was analyzed for the first time. These data represent an important source of information for public policy makers. The registry from which mortality data were obtained is of high quality, with an average proportion of 3% of deaths among individuals aged 35 years old or older coded as R99 of the ICD-10 (“Ill-defined and unspecified causes of mortality”). This proportion decreased from 4.1% in 2009 to 3.4% in 2018, followed by a slight increase to approximately 4% from 2019 onward^38^. In all years, this proportion remained below the 10% threshold commonly used as a reference for high-quality mortality records. Another strength of this study is the adherence to recommendations aimed at improving the quality of estimates when attributing mortality to a risk factor^23^.

Between 2009 and 2021, 4.8% of deaths from lung cancer, ischemic heart disease, CVD, COPD, type 2 diabetes mellitus, lower respiratory tract infections, asthma, and breast cancer among individuals aged 35 years old or older in Brazilian capitals were attributable to secondhand smoke, corresponding to approximately 5,000 deaths per year. Of these attributable deaths, 6 out of 10 were due to CVD. AM to secondhand smoke was higher among men (six out of ten attributable deaths) and varied across Brazilian capitals. Overall, AM to secondhand smoke in Brazil showed a favorable trend, with rates decreasing by approximately half over the study period.

Although Brazil is a global leader in tobacco control^2^, additional measures are still required to further reduce tobacco consumption and passive smoking. Key strategies include sustained increases in taxation, restrictions on points of sale, strengthened enforcement against smuggling and illicit tobacco trade, incentives for alternatives to tobacco cultivation, and the implementation of plain tobacco packaging^39^. Tobacco control policies in Brazil should be continuously monitored, expanded, and coordinated with intersectoral actions focused on equity and social justice, in alignment with the Sustainable Development Goals (SDGs), to reduce inequalities and enhance health gains. The continued mobilization of Brazilian civil society is essential for the protection and advancement of public policies, as advocated by WHO/FCTC.

## Supplementary material

Supplementary material
