# Supplementary material for: Evolution of mortality attributable to passive smoking in the 27 Brazilian capitals, 2009-2021
Source: Rev Bras Epidemiol. 2026 Apr 3;29:e260017. doi: 10.1590/1980-549720260017 (PMC13053034; doi:10.1590/1980-549720260017)

**MATERIAL SUPLEMENTAR**

**Tabela S1.** Mortalidade atribuível (MA) e taxas brutas (TB) por 100.000 habitantes de mortalidade atribuível ao tabagismo passivo nos homens de 35 e mais anos, por causas de morte nas 27 capitais do Brasil, 2009-2021

| **Capital** | **Câncer de pulmão** | | **Cardiopatia isquêmica** | | **Enfermidade cerebrovascular** | | **Doença pulmonar obstrutiva crônica** | | **Diabetes mellitus tipo 2** | | **Infecções do trato respiratório inferior** | | **Asma** | | **Câncer de mama** | |
|---|---|---|---|---|---|---|---|---|---|---|---|---|---|---|---|---|
| | **MA** | **TB** | **MA** | **TB** | **MA** | **TB** | **MA** | **TB** | **MA** | **TB** | **MA** | **TB** | **MA** | **TB** | **MA** | **TB** |
| Aracaju | 52 | 3,5 | 109 | 7,2 | 105 | 7,0 | 3 | 0,2 | 59 | 3,9 | 91 | 6,0 | 2 | 0,1 | 0 | 0,0 |
| Belém | 146 | 4,0 | 371 | 10,1 | 256 | 7,0 | 24 | 0,7 | 125 | 3,4 | 372 | 10,1 | 2 | 0,1 | 3 | 0,1 |
| Belo Horizonte | 312 | 4,4 | 428 | 6,1 | 318 | 4,5 | 11 | 0,2 | 144 | 2,0 | 436 | 6,2 | 7 | 0,1 | 6 | 0,1 |
| Boa Vista | 17 | 2,3 | 41 | 5,6 | 29 | 3,9 | 3 | 0,4 | 21 | 2,8 | 35 | 4,7 | 0 | 0,0 | 1 | 0,1 |
| Brasília | 160 | 2,2 | 345 | 4,8 | 227 | 3,2 | 16 | 0,2 | 106 | 1,5 | 217 | 3,0 | 6 | 0,1 | 1 | 0,0 |
| Campo Grande | 99 | 4,4 | 323 | 14,3 | 150 | 6,6 | 12 | 0,5 | 45 | 2,0 | 255 | 11,3 | 1 | 0,1 | 1 | 0,0 |
| Cuiabá | 67 | 4,3 | 143 | 9,2 | 78 | 5,0 | 18 | 1,2 | 55 | 3,5 | 99 | 6,4 | 2 | 0,1 | 1 | 0,1 |
| Curitiba | 193 | 3,7 | 431 | 8,2 | 178 | 3,4 | 9 | 0,2 | 138 | 2,6 | 134 | 2,6 | 4 | 0,1 | 2 | 0,0 |
| Florianópolis | 125 | 9,0 | 145 | 10,4 | 48 | 3,4 | 9 | 0,6 | 19 | 1,3 | 56 | 4,0 | 1 | 0,0 | 1 | 0,0 |
| Fortaleza | 283 | 4,5 | 518 | 8,2 | 391 | 6,2 | 16 | 0,3 | 122 | 1,9 | 520 | 8,3 | 7 | 0,1 | 3 | 0,1 |
| Goiânia | 197 | 5,2 | 415 | 11,0 | 226 | 6,0 | 9 | 0,2 | 91 | 2,4 | 300 | 7,9 | 2 | 0,0 | 3 | 0,1 |
| João Pessoa | 73 | 3,7 | 228 | 11,7 | 150 | 7,7 | 35 | 1,8 | 80 | 4,1 | 160 | 8,2 | 1 | 0,1 | 1 | 0,1 |
| Macapá | 20 | 2,1 | 42 | 4,5 | 39 | 4,1 | 15 | 1,7 | 25 | 2,7 | 39 | 4,2 | 1 | 0,1 | 1 | 0,1 |
| Maceió | 60 | 2,6 | 237 | 10,2 | 200 | 8,6 | 4 | 0,2 | 122 | 5,2 | 184 | 7,9 | 2 | 0,1 | 2 | 0,1 |
| Manaus | 141 | 3,2 | 242 | 5,4 | 197 | 4,4 | 5 | 0,1 | 151 | 3,4 | 219 | 4,9 | 3 | 0,1 | 3 | 0,1 |

| | | | | | | | | | | | | | | | | |
|---|---|---|---|---|---|---|---|---|---|---|---|---|---|---|---|---|
| Natal | 111 | 5,1 | 329 | 15,1 | 165 | 7,6 | 139 | 6,4 | 110 | 5,1 | 218 | 10,0 | 2 | 0,1 | 2 | 0,1 |
| Palmas | 16 | 2,8 | 39 | 6,9 | 35 | 6,2 | 3 | 0,6 | 21 | 3,7 | 28 | 4,9 | 0 | 0,1 | 0 | 0,1 |
| Porto Alegre | 422 | 10,1 | 467 | 11,2 | 256 | 6,1 | 8 | 0,2 | 155 | 3,7 | 190 | 4,5 | 6 | 0,1 | 3 | 0,1 |
| Porto Velho | 32 | 2,7 | 54 | 4,5 | 44 | 3,7 | 47 | 4,0 | 21 | 1,8 | 50 | 4,2 | 1 | 0,1 | 1 | 0,1 |
| Recife | 243 | 5,8 | 637 | 15,1 | 448 | 10,6 | 32 | 0,8 | 174 | 4,1 | 265 | 6,3 | 5 | 0,1 | 2 | 0,0 |
| Rio Branco | 24 | 3,0 | 38 | 4,7 | 39 | 4,9 | 6 | 0,8 | 22 | 2,7 | 57 | 7,1 | 0 | 0,0 | 0 | 0,1 |
| Rio de Janeiro | 912 | 4,8 | 2131 | 11,2 | 918 | 4,8 | 10 | 0,1 | 592 | 3,1 | 1854 | 9,7 | 14 | 0,1 | 8 | 0,0 |
| Salvador | 230 | 3,1 | 398 | 5,5 | 276 | 3,8 | 15 | 0,2 | 160 | 2,2 | 295 | 4,0 | 6 | 0,1 | 3 | 0,0 |
| São Luís | 87 | 3,6 | 226 | 9,3 | 159 | 6,5 | 26 | 1,0 | 106 | 4,4 | 168 | 6,9 | 2 | 0,1 | 1 | 0,0 |
| São Paulo | 1391 | 4,2 | 4156 | 12,6 | 1459 | 4,4 | 180 | 0,5 | 635 | 1,9 | 2911 | 8,8 | 27 | 0,1 | 9 | 0,0 |
| Teresina | 115 | 5,9 | 250 | 12,8 | 237 | 12,1 | 63 | 3,2 | 119 | 6,1 | 236 | 12,1 | 3 | 0,1 | 4 | 0,2 |
| Vitória | 57 | 5,9 | 72 | 7,5 | 61 | 6,3 | 18 | 1,8 | 24 | 2,5 | 20 | 2,1 | 1 | 0,1 | 1 | 0,1 |
| Total | 5582 | 4,4 | 12817 | 10,0 | 6691 | 5,2 | 735 | 0,6 | 3442 | 2,7 | 9407 | 7,3 | 107 | 0,1 | 65 | 0,1 |

**Tabela S2.** Mortalidade atribuível (MA) e taxas brutas (TB) por 100.000 habitantes de mortalidade atribuível ao tabagismo passivo nas mulheres de 35 e mais anos, por causas de morte nas 27 capitais do Brasil, 2009-2021

| **Capital** | **Câncer de pulmão** | | **Cardiopatia isquêmica** | | **Enfermidade cerebrovascular** | | **Doença pulmonar obstrutiva crônica** | | **Diabetes mellitus tipo 2** | | **Infecções do trato respiratório inferior** | | **Asma** | | **Câncer de mama** | |
|---|---|---|---|---|---|---|---|---|---|---|---|---|---|---|---|---|
| | **MA** | **TB** | **MA** | **TB** | **MA** | **TB** | **MA** | **TB** | **MA** | **TB** | **MA** | **TB** | **MA** | **TB** | **MA** | **TB** |
| Aracaju | 26 | 1,3 | 56 | 2,8 | 70 | 3,5 | 1 | 0,1 | 45 | 2,2 | 60 | 3,0 | 2 | 0,1 | 30 | 1,5 |
| Belém | 63 | 1,4 | 147 | 3,2 | 161 | 3,5 | 15 | 0,3 | 85 | 1,9 | 268 | 5,8 | 4 | 0,1 | 74 | 1,6 |
| Belo Horizonte | 127 | 1,4 | 209 | 2,3 | 216 | 2,4 | 9 | 0,1 | 102 | 1,1 | 276 | 3,1 | 14 | 0,2 | 165 | 1,9 |
| Boa Vista | 6 | 0,8 | 11 | 1,5 | 13 | 1,7 | 1 | 0,1 | 14 | 1,9 | 17 | 2,3 | 0 | 0,1 | 5 | 0,7 |
| Brasília | 82 | 1,0 | 171 | 2,0 | 160 | 1,9 | 10 | 0,1 | 76 | 0,9 | 160 | 1,9 | 9 | 0,1 | 84 | 1,0 |
| Campo Grande | 43 | 1,6 | 120 | 4,5 | 86 | 3,2 | 4 | 0,2 | 30 | 1,1 | 141 | 5,3 | 2 | 0,1 | 40 | 1,5 |
| Cuiabá | 30 | 1,7 | 61 | 3,5 | 49 | 2,8 | 4 | 0,2 | 38 | 2,2 | 65 | 3,7 | 1 | 0,1 | 33 | 1,9 |
| Curitiba | 87 | 1,3 | 169 | 2,6 | 112 | 1,7 | 14 | 0,2 | 84 | 1,3 | 87 | 1,4 | 5 | 0,1 | 89 | 1,4 |
| Florianópolis | 44 | 2,7 | 70 | 4,3 | 32 | 1,9 | 3 | 0,2 | 13 | 0,8 | 38 | 2,3 | 2 | 0,1 | 35 | 2,2 |
| Fortaleza | 170 | 2,1 | 263 | 3,2 | 256 | 3,1 | 10 | 0,1 | 95 | 1,2 | 368 | 4,5 | 11 | 0,1 | 121 | 1,5 |
| Goiânia | 89 | 1,9 | 169 | 3,7 | 134 | 2,9 | 15 | 0,3 | 62 | 1,3 | 189 | 4,1 | 3 | 0,1 | 85 | 1,9 |
| João Pessoa | 38 | 1,5 | 118 | 4,7 | 98 | 3,9 | 4 | 0,2 | 62 | 2,4 | 122 | 4,8 | 2 | 0,1 | 40 | 1,6 |
| Macapá | 9 | 0,9 | 19 | 1,9 | 25 | 2,5 | 2 | 0,2 | 17 | 1,7 | 31 | 3,1 | 1 | 0,1 | 7 | 0,7 |
| Maceió | 35 | 1,1 | 107 | 3,5 | 118 | 3,8 | 6 | 0,2 | 86 | 2,8 | 124 | 4,1 | 3 | 0,1 | 33 | 1,1 |
| Manaus | 54 | 1,1 | 78 | 1,6 | 100 | 2,0 | 7 | 0,1 | 82 | 1,6 | 115 | 2,3 | 3 | 0,1 | 48 | 1,0 |
| Natal | 61 | 2,2 | 170 | 6,1 | 108 | 3,9 | 4 | 0,1 | 91 | 3,3 | 160 | 5,7 | 3 | 0,1 | 60 | 2,1 |

| | | | | | | | | | | | | | | | | |
|---|---|---|---|---|---|---|---|---|---|---|---|---|---|---|---|---|
| Palmas | 7 | 1,1 | 17 | 2,8 | 22 | 3,6 | 1 | 0,2 | 16 | 2,6 | 17 | 2,8 | 1 | 0,1 | 7 | 1,1 |
| Porto Alegre | 207 | 3,8 | 275 | 5,0 | 210 | 3,8 | 33 | 0,6 | 116 | 2,1 | 147 | 2,7 | 12 | 0,2 | 140 | 2,5 |
| Porto Velho | 13 | 1,1 | 22 | 1,9 | 25 | 2,2 | 3 | 0,2 | 15 | 1,3 | 30 | 2,6 | 1 | 0,1 | 10 | 0,9 |
| Recife | 139 | 2,4 | 401 | 7,0 | 334 | 5,8 | 24 | 0,4 | 157 | 2,7 | 220 | 3,8 | 11 | 0,2 | 167 | 2,9 |
| Rio Branco | 13 | 1,5 | 16 | 1,8 | 22 | 2,4 | 2 | 0,2 | 14 | 1,5 | 32 | 3,5 | 0 | 0,0 | 7 | 0,8 |
| Rio de Janeiro | 498 | 2,0 | 1266 | 5,2 | 765 | 3,1 | 53 | 0,2 | 508 | 2,1 | 1662 | 6,8 | 30 | 0,1 | 564 | 2,3 |
| Salvador | 111 | 1,2 | 216 | 2,3 | 203 | 2,1 | 5 | 0,0 | 117 | 1,2 | 196 | 2,1 | 8 | 0,1 | 143 | 1,5 |
| São Luis | 45 | 1,4 | 113 | 3,6 | 118 | 3,7 | 6 | 0,2 | 82 | 2,6 | 132 | 4,2 | 3 | 0,1 | 43 | 1,4 |
| São Paulo | 643 | 1,6 | 1973 | 4,8 | 943 | 2,3 | 78 | 0,2 | 417 | 1,0 | 1813 | 4,4 | 36 | 0,1 | 680 | 1,7 |
| Teresina | 44 | 1,7 | 96 | 3,7 | 127 | 4,9 | 4 | 0,2 | 73 | 2,8 | 140 | 5,4 | 3 | 0,1 | 45 | 1,7 |
| Vitória | 26 | 2,2 | 41 | 3,4 | 46 | 3,8 | 2 | 0,1 | 20 | 1,6 | 17 | 1,4 | 2 | 0,2 | 38 | 3,1 |
| Total | 2711 | 1,7 | 6374 | 4,0 | 4553 | 2,8 | 319 | 0,2 | 2519 | 1,6 | 6628 | 4,1 | 170 | 0,1 | 2795 | 1,7 |

**Figura S1.** Taxas brutas de mortalidade atribuível ao tabagismo passivo por causas, em global e em função de sexo em Belém (Pará), 2009-2021

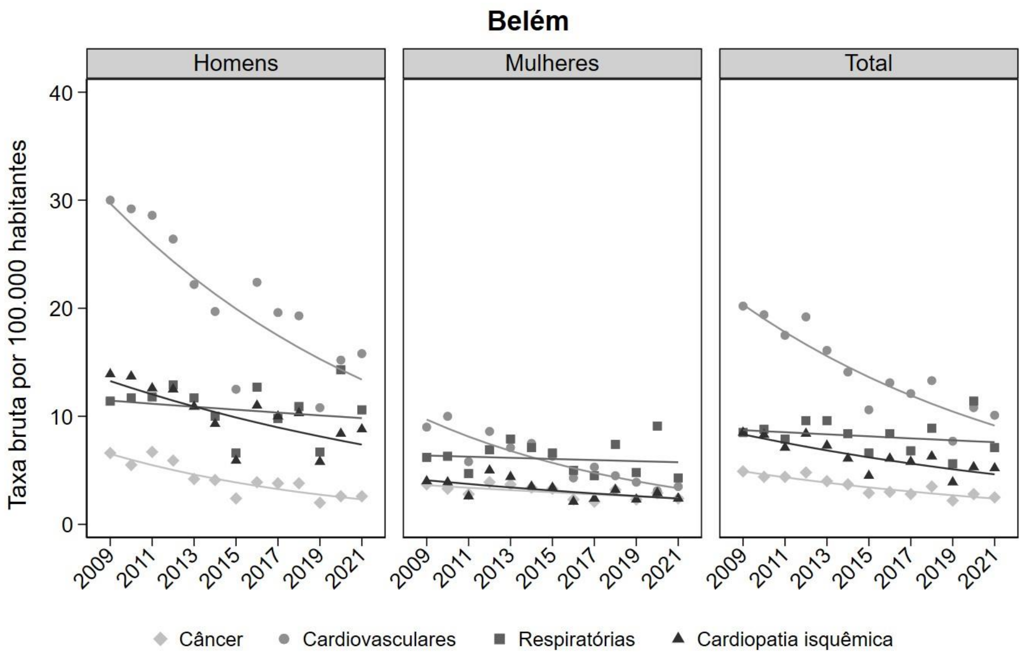


**Figura S2.** Taxas brutas de mortalidade atribuível ao tabagismo passivo por causas, em global e em função de sexo em Rio Branco (Acre), 2009-2021

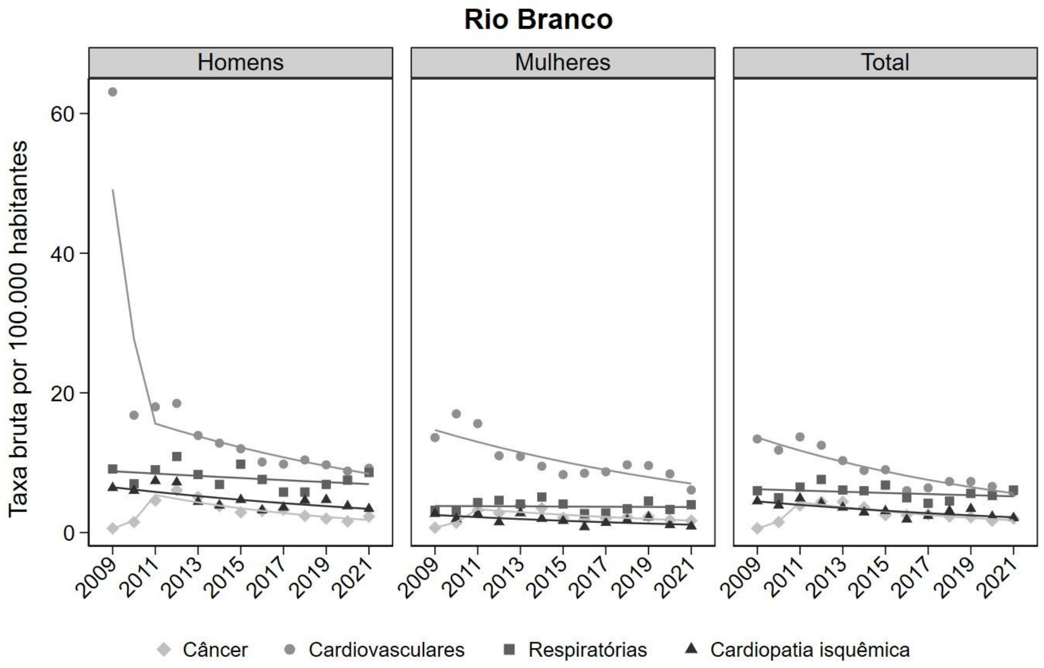

Supplement: Supplementary file 1 [file 1980-5497-rbepid-29-e260017-sppl.pdf]
