# Supplementary material for: Evolution of mortality attributable to passive smoking in the 27 Brazilian capitals, 2009-2021
Source: Rev Bras Epidemiol. 2026 Apr 3;29:e260017. doi: 10.1590/1980-549720260017 (PMC13053034; doi:10.1590/1980-549720260017)
Supplement: Translation

# Translation: Evolução da mortalidade atribuível ao tabagismo passivo nas 27 capitais brasileiras, 2009-2021

**DOI:** 10.1590/1980-549720260017.2

**Authors:** Bibiana Wanderlei Flores, Julia Rey-Brandariz, Paulo César Rodrigues Pinto Corrêa, Sofia Ravara, Agustín Montes Martinez, Mónica Pérez-Ríos

**Affiliations:** I Universidad de Santiago de Compostela, Departamento de Medicina Preventiva y Salud Pública - Santiago de Compostela, España.; II Consorcio para la Investigación Biomédica en Epidemiología y Salud Pública - Madrid, España.; III Universidade Federal de Ouro Preto, Escola de Medicina - Ouro Preto (MG), Brasil.; IV Universidade Federal de Minas Gerais, Faculdade de Ciências da Saúde - Belo Horizonte (MG), Brasil.; V Universidade da Beira Interior - Covilhã, Portugal.; VI Unidade Local de Saúde da Cova da Beira - Covilhã, Portugal.; VII Universidade NOVA de Lisboa, Escola Nacional de Saúde Pública, Centro de Investigação em Saúde Pública - Lisboa, Portugal.; VIII Instituto de Investigaciones Sanitarias de Santiago de Compostela - Santiago de Compostela, España.

**Keywords:** Tabagismo, Mortalidade, Brasil, Doenças cardiovasculares, Epidemiologia

## Abstract

**Objetivo:**

Estimar a mortalidade atribuível ao tabagismo passivo na população de 35 anos ou mais, por sexo, nas 27 capitais brasileiras, no período de 2009 a 2021.

**Métodos:**

Utilizou-se um método dependente de prevalência, baseado no cálculo de Frações Atribuíveis à População (FAP). As mortes por doenças causalmente relacionadas ao tabagismo passivo foram obtidas do Sistema de Informações sobre Mortalidade do Sistema Único de Saúde (SIM/SUS); a prevalência, dos inquéritos do Sistema de Vigilância de Fatores de Risco e Proteção para Doenças Crônicas por Inquérito Telefônico (Vigitel, 2009-2021); e os riscos relativos, de uma metanálise. A Mortalidade Atribuível (MA) ao tabagismo passivo e suas taxas foram estimadas por capital, ano, sexo e causas de morte. As tendências das taxas brutas de mortalidade atribuível ao tabagismo passivo foram analisadas aplicando modelos de regressão *joinpoint*.

**Resultados:**

O tabagismo passivo foi responsável por 64.913 mortes em todas as capitais brasileiras entre 2009 e 2021. As doenças cardiovasculares foram a principal causa de morte em ambos os sexos. A taxa de MA ao tabagismo passivo diminuiu de 33,1/100 mil mortes em 2009 para 15,4/100 mil mortes em 2021. Essa redução foi observada em todas as 27 capitais brasileiras, tanto de forma global quanto por sexo.

**Conclusão:**

O tabagismo passivo foi responsável por 1,4% de todas as mortes nas capitais brasileiras no período de 2009 a 2021 e apresentou uma tendência favorável, com as taxas diminuindo pela metade ao longo do período.

## INTRODUÇÃO

O tabagismo passivo constitui um importante problema de saúde pública global^1^, sendo responsável por aproximadamente 1,3 milhão de mortes anuais em todo o mundo^2^. Mais de 600 estudos científicos já demonstraram seus efeitos nocivos^3^, estabelecendo uma associação causal com o câncer de pulmão e a cardiopatia isquêmica em adultos^4^. Além disso, uma metanálise recente expandiu as evidências de causalidade para outras enfermidades, como a Doença Pulmonar Obstrutiva Crônica (DPOC), doença cerebrovascular, diabetes mellitus tipo 2, asma, as infecções do trato respiratório inferior e o câncer de mama^5^.

Os impactos do tabagismo passivo sobre a saúde impulsionaram a formulação de políticas públicas e estratégias populacionais efetivas, reunidas na Convenção-Quadro para o Controle do Tabaco da Organização Mundial de Saúde (CQCT/OMS) e no pacote MPOWER de 2008 da OMS^2^. No Brasil, embora a CQCT/OMS tenha sido ratificada em 2005, as medidas de proteção contra o tabagismo passivo começaram com a Lei n^o^ 9.294/1996^6^, e com suas alterações em 2011 (Lei n^o^ 12.546/2011)^7^ e 2014 (Decreto n^o^ 8.262/2014)^8^. Essas alterações incluíram a proibição nacional de publicidade, promoção e patrocínio de produtos de tabaco; o fortalecimento de programas de cessação com oferta de tratamento na atenção básica do sistema de saúde público brasileiro (SUS); e a proibição do consumo de derivados do tabaco em ambientes coletivos, públicos ou privados^7^ ^,^ ^8^. Para avaliar o impacto dessas medidas, é essencial estimar a prevalência e a Mortalidade Atribuível (MA) ao tabagismo ativo e passivo, bem como verificar possíveis mudanças ao longo do tempo.

A primeira estimativa da prevalência do tabagismo passivo no Brasil foi conduzida em 1989, por meio da Pesquisa Nacional de Saúde e Nutrição, que identificou uma prevalência de 31,7% entre adultos^9^. Em 2003, foi realizado o Inquérito Domiciliar sobre Comportamentos de Risco e Morbidade Referida de Doenças e Agravos Não Transmissíveis. De acordo com os dados dessa pesquisa, nas 27 capitais brasileiras, a prevalência do tabagismo passivo variou de 13%, em Campo Grande, a 26%, em Recife^10^. Desde 2009, o Sistema de Vigilância de Fatores de Risco e Proteção para Doenças Crônicas por Inquérito Telefônico (Vigitel) divulga anualmente a prevalência do tabagismo passivo nas capitais brasileiras. Entre 2009 e 2023, observou-se uma redução significativa dessa prevalência entre adultos com 18 anos ou mais, passando de 12,7% para 6,4% em casa, e de 12,1% para 7% no trabalho^11^ ^,^ ^12^.

No Brasil, 8 estudos estimaram a MA ao tabagismo passivo^13^ ^,^ ^14^ ^,^ ^15^ ^,^ ^16^ ^,^ ^17^ ^,^ ^18^ ^,^ ^19^ ^,^ ^20^; entretanto, nenhum deles analisou essa mortalidade por unidade federativa ou capital, tampouco considerou doenças recentemente associadas a esta exposição^5^. O Brasil é um país de dimensão continental e apresenta significativas desigualdades demográficas, socioeconômicas e epidemiológicas. Assim, estimativas regionais detalhadas são essenciais para avaliar o impacto das políticas de ambientes livres de fumo, reforçar sua efetividade e orientar adaptações conforme as necessidades locais, especialmente entre grupos populacionais mais vulneráveis.

Desse modo, o objetivo deste estudo foi estimar a MA ao tabagismo passivo nas 27 capitais brasileiras, na população de 35 anos ou mais, por sexo, no período de 2009 a 2021.

## MÉTODOS

### Delineamento e contexto do estudo

Trata-se de um estudo de mortalidade atribuível ao tabagismo passivo nas 27 capitais brasileiras nos anos 2009-2021. O Brasil é um país composto por 5 grandes regiões: Norte, Nordeste, Centro-Oeste, Sudeste e Sul. Estas são divididas em 27 unidades federativas, sendo que cada uma delas possui uma capital. Em 2024, a população brasileira foi estimada em 212.583.750, dos quais 23,1% (49.175.449) residiam nas capitais^21^.

### Métodos estatísticos

Para estimar a MA ao tabagismo passivo foi aplicado um método dependente de prevalências, baseado no cálculo das Frações Atribuíveis Populacionais (FAP)^22^. A estimativa foi realizada de acordo com as recomendações da guia STREAMS-p^23^.

A FAP foi calculada utilizando a seguinte fórmula:

$$FAP=\frac{\left(q+pxRR\right)-1}{q+pxRR}$$

FAP é a fração atribuível populacional; p é a prevalência ao tabagismo passivo; q=1-p; e RR é o risco de morrer dos indivíduos expostos ao tabagismo passivo, em comparação aos não expostos.

A MA é obtida multiplicando-se a FAP pela Mortalidade Observada (MO):

MA=FAPxMO

### Fontes de dados e população do estudo

A fonte de dados de mortalidade para a população em estudo foi a base de registro de óbitos do Sistema de Informação de Mortalidade do Sistema Único de Saúde do Brasil (SIM/SUS). Os dados de cada ano foram extraídos por sexo e codificados, segundo a 10^a^ edição da Classificação Internacional de Doenças (CID-10), por capital para as seguintes causas: câncer de pulmão, cardiopatia isquêmica, doença cerebrovascular, DPOC, diabetes mellitus tipo 2, infecções do trato respiratório inferior, asma e câncer de mama^5^. Para apresentar os resultados, as causas foram agrupadas em: câncer, doenças cardiovasculares (DCV) e doenças respiratórias.

A prevalência do tabagismo passivo para a população em estudo foi calculada por sexo e para cada uma das 27 capitais brasileiras, baseada nos microdados do Vigitel, produzidos entre 2009 e 2021. O Vigitel é um estudo transversal de base populacional que avalia a população adulta (>18 anos) residente nas capitais brasileiras. A amostra para estimar a prevalência de qualquer fator é determinada considerando um intervalo de confiança de 95% e um erro amostral de 2%, obtendo-se um tamanho mínimo de 2 mil entrevistas por capital de acordo com dois estágios: sorteio de domicílios com linha telefônica fixa e sorteio de um morador adulto para responder ao inquérito. No caso do tabagismo passivo, o inquérito é realizado com as seguintes perguntas: “Alguma das pessoas que moram com o(a) Sr.(a) costuma fumar dentro de casa?” e “Algum colega do trabalho costuma fumar no mesmo ambiente onde o(a) Sr.(a) trabalha?”. As opções de resposta são “sim” ou “não”. Considerou-se como exposta a pessoa que respondeu “sim” em qualquer uma das duas perguntas.

Os riscos de morrer associados ao tabagismo passivo foram obtidos do estudo de Flor et al.^5^: cardiopatia isquêmica [1,26 (1,20-1,32)], câncer de pulmão [1,37 (1,30-1,45)], doença cerebrovascular [1,16 (1,11-1,22)], DPOC [1,44 (1,21-1,71)], diabetes mellitus tipo 2 [1,16 (1,09-1,24)], infecções do trato respiratório inferior [1,34 (1,23-1,45)], asma [1,21 (1,16-1,26)] e câncer de mama [1,22 (1,13-1,31)].

### Análise estatística

Foram estimadas a MA e as respectivas taxas brutas por tabagismo passivo em cada capital brasileira em cada ano do período estudado (2009-2021), em função de sexo e causa de morte. Foram calculados Intervalos de Confiança de 95% (IC95%) para as taxas brutas, aplicando-se a seguinte fórmula^24^:

(100.000/p) (MA±(1,96 x √MA)

Onde p representa o denominador da taxa bruta, ou seja, a população utilizada para seu cálculo; e MA corresponde ao numerador, isto é, o número de mortes atribuídas considerado no cálculo da taxa bruta.

A tendência das taxas brutas de MA foi analisada aplicando modelos de regressão *joinpoint*. Inicialmente, fixou-se o máximo de 3 pontos de corte com base no Critério de Informação Bayesiano (BIC)^25^ e um nível de significância de 5%. Calculou-se a Variação Percentual por Ano (VPA) com intervalos de confiança de 95% (IC95%). A estimativa de MA foi realizada com o programa Stata v. 17^26^ e os modelos de regressão *joinpoint*, com o programa *Joinpoint Regression Program* v. 5.3^27^.

### Aspectos éticos

Este estudo utilizou dados secundários, os quais estão disponíveis para acesso e uso público. A Resolução n^o^ 466/2012^28^, que trata de pesquisa envolvendo seres humanos, estabelece que dados de domínio público que não envolvam a identificação ou possibilidade de identificação dos participantes não necessitam de aprovação de um Comitê de Ética em Pesquisa (CEP).

## Declaração de disponibilidade dos dados

O banco de dados e os códigos de análise utilizados na pesquisa estão disponíveis em https://svs.aids.gov.br/download/Vigitel/

## RESULTADOS

No período de 2009-2021, o tabagismo passivo foi responsável por 64.913 mortes na população de 35 anos ou mais nas capitais brasileiras, correspondendo a 4,8% da mortalidade observada pelas causas em estudo e 1,4% da mortalidade observada por todas as causas no mesmo período. Do total de mortes atribuíveis ao tabagismo passivo, 59,8% (38.845) ocorreram em homens, sendo 56,1% (36.396) devido às Doenças Cardiovasculares (DCV). A cardiopatia isquêmica foi a principal causa específica de mortalidade em homens (12.817 mortes; 33,6% da mortalidade atribuível total), enquanto nas mulheres foram as infecções do trato respiratório inferior (6.628 mortes; 25,4% da MA total) (Tabelas Suplementares 1 e 2).

As taxas brutas de MA ao tabagismo passivo diminuíram em todas as capitais brasileiras no período estudado, passando de 33,1 mortes por 100 mil habitantes em 2009 para 15,4 em 2021. Essa redução foi observada em ambos os sexos. Entre os homens, as taxas diminuíram de 27,7 mortes por 100 mil habitantes em 2009 para 12,8 em 2021. Já entre as mulheres, as taxas diminuíram de 13,4 para 5,2 mortes por 100 mil habitantes no mesmo período (Figura 1). Globalmente, essa diminuição foi mais acentuada entre 2009 e 2016 (VPA -7,8; IC95% de -10,6 a -5). Em homens, observamos 3 períodos: o primeiro, com tendência decrescente até 2016 (VPA de -8; IC95% de -11 a -4,2); o segundo, com tendência estável até 2019 (VPA de 0,5; IC95% de -9,6 a 3,3); e o terceiro, com tendência decrescente entre 2019 e 2021 (VPA de -9,2; IC95% de -14,2 a -2,4). Em mulheres, desde 2009, a tendência das taxas de MA foi sempre decrescente (VPA de -6,6; IC95% de -7,5 a -5,7). As VPAs e os IC 95% são apresentados na Tabela 1.

**Figura 1.** Taxas brutas de mortalidade atribuível ao tabagismo passivo e sua tendência, por todas as causas, total e por sexo, nas 27 capitais brasileiras, 2009-2021.

**Tabela 1.** Variação Percentual por Ano e intervalo de confiança de 95% por sexo nas 27 capitais brasileiras, 2009-2021.

Capital	VPA (IC95%)		
	2009-2021		
	Global	Homens	Mulheres
Aracaju	-9,7 (-11,8 a -7,5)	-9,7 (-13,2 a -6)	-9,6 (-12,4 a -6,8)
Belém	-4,6 (-6,8 a -2,3)	-5 (-8,2 a -1,5)	-3,8 (-9,4 a 1,9)
Belo Horizonte	-6,3 (-9,2 a -3,2)	-5,3 (-7,7 a -2,8)	-7,9 (-10,7 a -5)
Boa Vista	-5 (-7,1 a -2,8)	-4,1 (-6,2 a -1,9)	-7,2 (-10,6 a -3,7)
Brasília	-6,1 (-7,7 a -4,4)	-6,2 (-8,6 a -3,6)	-6,3 (-9,3 a -3,3)
Campo Grande	-3,2 (-4,9 a -1,3)	-2,6 (-5,3 a 0,2)	-4,2 (-6,4 a -1,9)
Cuiabá	-8,4 (-12,2 a -4,3)	-7,1 (-10,8 a -3,3)	-10,6 (-14,8 a -6,2)
Curitiba	-8,8 (-11,8 a -5,7)	-8,8 (-11,2 a -6,2)	-8,9 (-12,8 a -4,7)
Florianópolis	-5,5 (-8,2 a -2,7)	-5 (-9,4 a -0,4)	-6,6 (-9,5 a -3,6)
Fortaleza	-4,7 (-7 a -2,3)	-3,9 (-6,3 a -1,5)	-5,7 (-7,2 a -4,1)
Goiânia	-9,0 (-10,6 a -7,4)	-8,3 (-10,8 a -5,7)	-10,1 (-11,4 a -8,7)
João Pessoa	-6,9 (-9,6 a -4,2)	-6,7 (-8,1 a -4,3)	-8 (-11,6 a -4,5)
Macapá	-2,1 (-5,1 a 0,9)	-2,5 (-5,7 a 0,7)	-1,4 (-3,1 a 0,2)
Maceió	-7,2 (-10,6 a -3,8)	-6,1 (-10,6 a -1,4)	-8,9 (-12,8 a -4,7)
Manaus	-3,8 (-5,9 a -1,7)	-2,9 (-6,7 a 0,9)	-5,6 (-8,2 a -2,9)
Natal	-5,8 (-8,3 a -3,3)	-6,2 (-8,8 a -3,5)	-5,3 (-7,3 a -3,3)
Palmas	-3,9 (-7,7 a 0,1)	-3,0 (-9 a 3,3)	-5,8 (-9,2 a -2,2)
Porto Alegre	-6,8 (-8,6 a -5)	-6,1 (-8,9 a -3,3)	-7,6 (-9,8 a -5,4)
Porto Velho	-8,9 (de -10,9 a -6,7)	-9,8 (-12 a -7,6)	-7,5 (-11,4 a -3,3)
Recife	-6,4 (-9,5 a -3,1)	-5,8 (-8 a -3,4)	-7,1 (-9,9 a -4,2)
Rio Branco	-5 (de -9 a -0,9)	-4,7 (-7,5 a -1,7)	-4,7 (-7,5 a -1,7)
Rio de Janeiro	-5 (-8,1 a -1,8)	-5,5 (-9,7 a -1,1)	-4,5 (-7,7 a -1,3)
Salvador	-5,1 (-6,6 a -3,5)	-4,7 (-6,4 a -3)	-5,5 (-8,6 a -2,3)
São Luís	-4,6 (-5,8 a -3,3)	-3,7 (-5,4 a -1,9)	-5,5 (-7,4 a -3,6)
São Paulo	-6,4 (-8 a -4,9)	-5,9 (-7,9 a -3,9)	-7,6 (-9,8 a -5,2)
Teresina	-7 (-8,9 a -5)	-6,9 (-9,4 a -4,4)	-6,9 (-9,7 a -3,9)
Vitória	-4,3 (-7,4 a -1)	-2,9 (-7,3 a 1,6)	-6,4 (-10 a -2,6)

VPA: Variação Percentual por Ano; IC95%: intervalo de confiança de 95%.

A Tabela 2 apresenta as taxas brutas de MA ao tabagismo passivo (por 100 mil habitantes) nas capitais brasileiras em 2009 e 2021. Recife/PE registrou a maior diminuição, passando de 48,4 em 2009 para 17,7 em 2021. A menor diminuição foi observada em Palmas (TO), onde as taxas variaram de 26,8 para 22,6 no mesmo período. Entre os homens, a taxa de MA ao tabagismo passivo diminuiu em 26 capitais, com exceção de Palmas, onde aumentou de 34,2 em 2009 para 39,9 em 2021. Entre as mulheres, observou-se queda nas taxas em todas as capitais.

**Tabela 2.** Taxas brutas de mortalidade atribuível ao tabagismo passivo (por 100 mil habitantes) e intervalo de confiança de 95%, por sexo, nas 27 capitais brasileiras, 2009-2021.

Capital	Taxa bruta de mortalidade atribuível (IC95%)					
	2009			2021		
	Global	Homens	Mulheres	Global	Homens	Mulheres
Aracaju	33,9 (26,2 a 41,6)	39,6 (27 a 52,2)	29,5 (19,9 a 39,1)	12 (8,2 a 15,7)	18,6 (11,4 a 25,8)	7 (3,2 a 10,8)
Belém	33,6 (28,7 a 38,5)	47,8 (39,1 a 56,6)	22 (16,7 a 27,4)	19,7 (16,5 a 22,9)	28,9 (23,1 a 34,8)	12,4 (9 a 15,8)
Belo Horizonte	22,6 (19,8 a 22,5)	29,1 (24,2 a 33,9)	17,5 (14,2 a 20,9)	13,8 (11,8 a 15,7)	22,2 (18,5 a 26)	7,1 (5,3 a 9)
Boa Vista	17,9 (8,8 a 27,1)	24,5 (9,2 a 39,7)	11,5 (1,1 a 21,9)	11,1 (6 a 16,1)	15,6 (7,1 a 24)	6,6 (1,1 a 12,1)
Brasília	18,3 (15,6 a 21,1)	22,9 (18,4 a 27,4)	14,5 (11,2 a 17,8)	8,1 (6,6 a 9,5)	8,8 (6,6 a 11)	7,5 (5,6 a 9,4)
Campo Grande	34,1 (27,6 a 40,5)	45,8 (34,9 a 56,8)	23,9 (16,6 a 31,3)	25,4 (20,7 a 30,1)	38,5 (30 a 47,1)	14,5 (9,7 a 19,3)
Cuiabá	33,4 (25,6 a 41,2)	39 (26,7 a 51,3)	28,3 (18,4 a 38,2)	7 (4 a 10)	10,6 (5,2 a 15,9)	3,9 (0,8 a 7)
Curitiba	27,5 (23,8 a 31,2)	38,8 (32,2 a 45,3)	18,2 (14,2 a 22,3)	11,2 (9,2 a 13,3)	14,1 (10,6 a 17,5)	8,9 (6,4 a 11,4)
Florianópolis	31,1 (23,1 a 39,1)	42,5 (28,6 a 56,3)	21,5 (12,5 a 30,6)	13,6 (9,3 a 17,9)	19,5 (12 a 27,1)	8,5 (3,9 a 13,2)
Fortaleza	27,9 (24,6 a 31,3)	35,4 (29,7 a 41,1)	22,1 (18,1 a 26)	15,5 (13,4 a 17,7)	21,4 (17,6 a 25,3)	11 (8,6 a 13,4)
Goiânia	41,3 (35,8 a 46,8)	60,6 (50,7 a 70,5)	25,3 (19,4 a 31,1)	12,5 (10 a 15)	18,6 (14,1 a 23,1)	7,4 (4,8 a 10)
João Pessoa	36,8 (29,8 a 43,8)	48,2 (36,1 a 60,3)	28 (19,9 a 36,1)	13,2 (9,6 a 16,7)	20,4 (13,7 a 27,1)	7,7 (4,1 a 11,3)
Macapá	18,3 (10,3 a 26,3)	25,4 (11,9 a 38,9)	11,6 (2,7 a 20,4)	13,4 (8,2 a 18,6)	16 (7,8 a 24,3)	10,9 (4,5 a 17,4)
Maceió	35 (28,8 a 41,2)	44,4 (33,9 a 54,9)	27,7 (20,4 a 35)	13,5 (10,2 a 16,8)	18,7 (12,8 a 24,7)	9,6 (5,9 a 13,3)
Manaus	24,1 (20,1 a 28,2)	32,7 (25,9 a 39,6)	16,3 (11,7 a 20,9)	14,7 (12,2 a 17,2)	20,7 (16,3 a 25)	9,4 (6,7 a 12,2)
Natal	52,2 (44,3 a 60)	71,3 (57,4 a 85,2)	37,3 (28,5 a 46,2)	22,8 (18,4 a 27,3)	30,1 (22,4 a 37,8)	17,1 (12 a 22,2)
Palmas	26,8 (14,2 a 39,5)	34,2 (14,2 a 54,2)	19,3 (4,1 a 34,5)	22,6 (14 a 31,2)	39,9 (23,2 a 56,6)	7,7 (0,8 a 14,5)
Porto Alegre	40,2 (35,4 a 45)	52,6 (44,3 a 61)	30,9 (25,3 a 36,4)	15,3 (12,6 a 18)	21,4 (16,6 a 26,2)	10,6 (7,6 a 13,6)
Porto Velho	25,4 (17 a 33,8)	37,8 (23,4 a 52,2)	12,8 (4,4 a 21,3)	8,3 (4,5 a 12)	11,3 (5,2 a 17,4)	5 (0,9 a 9,2)
Recife	48,4 (43,2 a 53,7)	61,3 (52,2 a 70,4)	38,9 (32,7 a 45,1)	17,7 (14,9 a 20,6)	26,1 (20,9 a 31,4)	11,6 (8,6 a 14,6)
Rio Branco	20,1 (11,4 a 28,7)	28,4 (13,5 a 43,4)	12,5 (3,1 a 21,9)	13,9 (8,2 a 19,6)	19,2 (9,3 a 29,1)	9,3 (2,8 a 15,7)
Rio de Janeiro	38,9 (36,7 a 41,4)	56,6 (52,5 a 60,7)	25,1 (22,8 a 27,5)	21,8 (20,3 a 23,3)	31,6 (28,9 a 34,4)	14,1 (12,4 a 15,7)
Salvador	21,1 (18,4 a 23,9)	26,8 (22,2 a 31,5)	16,7 (13,4 a 19,9)	11,3 (9,6 a 13)	16,8 (13,7 a 20)	7,1 (5,3 a 8,9)
São Luís	32,9 (26,9 a 38,9)	47,4 (36,4 a 58,2)	21,3 (14,8 a 27,7)	18,4 (14,7 a 22,2)	24,7 (18,1 a 31,3)	13,7 (9,5 a 18)
São Paulo	34,7 (33 a 36,3)	46,2 (43,4 a 49,1)	25,3 (23,5 a 27,2)	14,5 (13,6 a 15,4)	19,5 (17,9 a 21,1)	10,5 (9,4 a 11,5)
Teresina	48 (40,1 a 55,9)	72,8 (58,2 a 87,5)	28,6 (20,5 a 36,7)	21,9 (17,3 a 26,5)	31,2 (22,8 a 39,7)	15,1 (10,1 a 20,1)
Vitória	38,9 (28,6 a 49,1)	46,6 (29,7 a 63,4)	32,7 (20,1 a 45,3)	19,7 (13,5 a 26)	31 (19,3 a 42,7)	10,7 (4,6 a 16,9)

IC95%: intervalo de confiança de 95%.

A análise por grandes grupos de causas de morte revela que as DCV apresentaram as taxas mais altas durante todo o período estudado, em todas as capitais brasileiras (Figura 2). Recife/PE foi a capital que apresentou as maiores taxas de MA ao tabagismo passivo por DCV ao longo de todo o período (Figura 3). Cabe ressaltar que em Belém (PA) as DCV foram a principal causa de morte até 2020; e em Rio Branco/AC até 2021, quando foram superadas pelas doenças respiratórias (Figuras Suplementares S1 e S2).

**Figura 2.** Taxas brutas de mortalidade atribuível ao tabagismo passivo, por causas, por sexo, nas 27 capitais brasileiras, 2009-2021.

**Figura 3.** Taxas brutas de mortalidade atribuível ao tabagismo passivo, por causas, total e por sexo, em Recife (PE), 2009-2021.

## DISCUSSÃO

No período de 2009-2021 o tabagismo passivo foi responsável por 64.913 mortes nas 27 capitais brasileiras; 6 em cada 10 ocorreram em homens, sendo atribuídas principalmente a DCV. A principal causa específica de morte relacionada ao tabagismo passivo foi a cardiopatia isquêmica nos homens, e as infecções do trato respiratório inferior nas mulheres. Entre 2009 e 2021, a MA ao tabagismo passivo reduziu significativamente: de 6.290 para 3.931 mortes.

A diminuição da MA ao tabagismo passivo ocorreu em todas as capitais, embora com magnitudes distintas, possivelmente relacionadas a fatores socioeconômicos. Recife (PE) apresentou a maior redução em ambos os sexos. Estudo prévio conduzido no Brasil também apontou Pernambuco como a unidade federativa com maior queda nas taxas de MA ao tabaco entre 1990 e 2017^29^. Palmas (TO), Macapá (AP), Rio Branco (AC) e Boa Vista (RR) apresentaram a menor diminuição de taxas de MA. Essas capitais possuem um Índice de Desenvolvimento Humano (IDH) abaixo da média nacional^30^. O IDH é um indicador que avalia o nível de desenvolvimento de uma população a partir de três dimensões fundamentais: educação, renda e longevidade^31^. Estudo realizado no Brasil em 2011 mostrou maior prevalência do tabagismo passivo em casa e no trabalho entre pessoas de baixa renda e menor escolaridade^32^, destacando a importância de abordar as desigualdades no combate ao consumo de tabaco e de fortalecer as políticas e os programas existentes^33^.

Em 2021, Campo Grande (MS) e Natal (RN) apresentaram as maiores taxas de MA ao tabagismo passivo. Campo Grande destaca-se por apresentar a maior prevalência de consumo de tabaco e está entre as dez capitais com a maior prevalência do tabagismo passivo em casa^11^. Já Natal, situada em uma unidade federativa com IDH inferior à média nacional, apresenta limitações no acesso aos serviços de saúde, o que pode comprometer a efetividade das políticas de controle do tabaco e contribuir para maiores taxas de mortalidade^30^. Em contraste, Cuiabá (MT) e Brasília (DF) apresentaram as menores taxas de MA. Ambas as capitais possuem elevado IDH, condição associada à melhor implementação das políticas de controle do tabaco e menor prevalência de consumo e de tabagismo passivo^30^. Em Cuiabá (MS), a prevalência do tabagismo passivo em casa está entre as mais baixas do país^11^.

Três estudos prévios que estimaram a MA ao tabagismo passivo no Brasil utilizando a metodologia do *Global Burden of Disease* (GBD)^18^ ^,^ ^19^ ^,^ ^20^ reportam taxas de MA superiores às deste estudo: 44,5/100 mil habitantes (18) e 32,8/100 mil habitantes^19^, em comparação com 19,2/100 mil habitantes observadas em nosso estudo. Outro trabalho recente^20^ apresenta os resultados por sexo: 14,2/100 mil habitantes nos homens e 6/100 mil habitantes nas mulheres, em comparação a 3,7/100 mil habitantes nos homens e 1,5/100 mil habitantes nas mulheres, conforme apontado por nosso estudo. Apesar de a metodologia neste estudo ser a mesma que a utilizada nos estudos do GBD, existem diferenças no processo de cálculo, causas incluídas, faixas etárias e abrangência geográfica. A metodologia do GBD ajusta as mortes não codificadas e redistribui os denominados “*Códigos Garbage*” (CG), o que não foi aplicado no presente estudo devido à baixa proporção desses códigos no Brasil (<5%).

Malta et al. analisaram o efeito da redistribuição dos CG na evolução da mortalidade por doenças crônicas no Brasil (2010-2019), comparando dados brutos do SIM, dados corrigidos por CG e sub-registro, e dados do GBD. Apesar de pouca alteração nas tendências temporais, as taxas corrigidas foram significativamente maiores, com variação heterogênea entre as unidades federativas^34^. Em relação às causas de morte, incluímos 8: cardiopatia isquêmica, doença cerebrovascular, câncer de pulmão, câncer de mama, infecções respiratórias inferiores, diabetes tipo 2, DPOC e asma. Reitsma et al.^19^ excluem a asma; São José et al.^18^ analisam apenas câncer; e Salerno et al.^20^, somente câncer de pulmão. A faixa etária populacional também varia: 35 anos ou mais no presente estudo; 40 anos ou mais no estudo de São José et al.^18^; 25 anos ou mais no de Reitsma et al.^19^; e 20 anos ou mais na pesquisa de Salerno et al.^20^ Difere-se, ainda, de São José et al.^18^, que consideraram a carga conjunta do tabagismo ativo e passivo. Além disso, é importante destacar que o presente estudo se limita à população das capitais brasileiras, representando aproximadamente 23% da população total do país, enquanto os demais abrangem a população nacional.

Conforme estudos anteriores no Brasil^15^ ^,^ ^16^, a MA ao tabagismo passivo é maior em homens. Essa diferença pode ser explicada pela maior prevalência do tabagismo passivo no ambiente de trabalho entre homens (10,2%), mais que o dobro da observada entre mulheres (4,3%)^11^. É importante destacar que as diferenças na MA ao tabagismo passivo - tanto no geral, quanto por causa específica e por sexo (cardiopatia isquêmica nos homens e doenças respiratórias nas mulheres) - também foram descritas em um outro estudo do nosso grupo sobre MA ao consumo de tabaco no Brasil^35^.

As estimativas de MA ao tabagismo passivo na América do Sul são escassas^36^. Um estudo no Chile estimou 70 mortes por câncer de pulmão atribuíveis ao tabagismo passivo^37^. Aplicando a mesma metodologia, o autor estimou 387 mortes no Brasil^15^. Estudo recente mostrou disparidades nas taxas de MA por câncer de traqueia, brônquios e pulmão: o Uruguai apresentou as maiores taxas entre homens (42,6/100 mil habitantes) e a Argentina, entre mulheres (8,8/100 mil habitantes). O Peru teve as menores taxas em ambos os sexos (3,6 e 0,7/100 mil habitantes). O Brasil ocupou posição intermediária, com a 6^a^ menor taxa entre homens (14,2/100 mil habitantes) e a 3^a^ entre mulheres (6/100 mil habitantes)^20^.

Este estudo possui limitações relacionadas ao método de estimação e às fontes de dados utilizadas. Na estimativa da MA, não foi considerado o tempo de latência entre o tabagismo passivo e a mortalidade, uma vez que ambos os indicadores são concorrentes no tempo. Esse fato decorre da ausência de informações específicas sobre os períodos de latência, que provavelmente variam conforme a causa e o nível de exposição. Em termos de prevalência, o Vigitel se baseia em informações de exposição autorrelatadas, o que pode levar a uma subestimação da prevalência do tabagismo passivo devido ao viés de memória. Há também a possibilidade de viés de seleção, pois a amostra é selecionada de um diretório de linhas telefônicas, o que exclui pessoas que não têm esse serviço, embora pesos estatísticos pós-estratificação sejam aplicados para minimizar esse efeito. No que diz respeito à mortalidade observada, os CG (aproximadamente 5% da mortalidade) não foram redistribuídos, pois isso acrescentaria incerteza às estimativas apresentadas, o que sugere que as estimativas de MA possivelmente subestimam a carga real da doença, embora se presuma que tal subestimação seja de pequena magnitude.

É importante considerar que a pandemia de COVID-19 pode ter influenciado tanto a qualidade dos registros de mortalidade quanto a codificação das causas de morte em 2020 e 2021. O excesso de mortes e a ausência inicial de um código específico para a covid-19 nos registros podem ter levado à codificação incorreta de mortes. Devido à ausência de estudos nacionais que estimem Riscos Relativos (RR) específicos para a associação entre tabagismo passivo e mortalidade no Brasil, optou-se pela utilização dos RR de uma metanálise recente^5^. Adicionalmente, embora reconhecidas as possíveis variações na distribuição etária das populações residentes nas capitais brasileiras ao longo dos 13 anos analisados, as taxas de mortalidade não foram ajustadas, uma vez que o objetivo deste estudo é avaliar a carga de MA de forma global, sem considerar modificações na estrutura etária.

Este estudo também apresenta pontos fortes significativos: pela primeira vez, a MA ao tabagismo passivo foi estimada por causas mais recentes utilizando prevalências representativas por sexo, além do mesmo processo de cálculo e as mesmas fontes de dados em todas as capitais brasileiras, permitindo uma comparação direta dos resultados. Também, foi analisada pela primeira vez a evolução da MA ao tabagismo passivo nas capitais brasileiras. Esses dados representam uma importante fonte de informação para formuladores de políticas públicas. O registro de onde procedem os dados de mortalidade apresenta alta qualidade, com proporção média de 3% de óbitos entre pessoas com 35 anos ou mais codificados como R99 da CID-10 (“Causas mal definidas e não especificadas de mortalidade”). Houve redução de 4,1% em 2009 para 3,4% em 2018, seguida de leve aumento para cerca de 4% a partir de 2019^38^. Em todos os anos a proporção permaneceu abaixo do limite de 10%, utilizado como referência para registros de mortalidade de alta qualidade. Outro ponto forte deste estudo é que foram seguidas recomendações destinadas a melhorar a qualidade das estimativas na atribuição da mortalidade a um fator de risco^23^.

Entre 2009 e 2021, 4,8% das mortes por câncer de pulmão, cardiopatia isquêmica, doença cerebrovascular, DPOC, diabetes tipo 2, infecções respiratórias inferiores, asma e câncer de mama em pessoas com 35 anos ou mais nas capitais brasileiras foram atribuíveis ao tabagismo passivo, contabilizando aproximadamente 5 mil mortes por ano. Das mortes atribuíveis, 6 em cada 10 foram causadas por doenças cardiovasculares. A MA ao tabagismo passivo é maior entre homens (seis em cada dez mortes atribuíveis) e varia entre as capitais brasileiras. Pode-se concluir que a MA ao tabagismo passivo no Brasil mostra uma evolução favorável, com as taxas de MA reduzindo rapidamente pela metade no período analisado.

Embora o Brasil seja referência mundial no controle do tabagismo^2^, ainda há medidas a serem implementadas para reduzir o consumo de tabaco e o tabagismo passivo. Destacam-se o aumento sustentado de impostos, a restrição de pontos de venda, o reforço da fiscalização do contrabando e do comércio ilícito de tabaco, o incentivo a alternativas ao cultivo de tabaco e a implementação de embalagens genéricas de tabaco (*plain tobacco packaging*)^39^. As políticas de controle do tabaco no Brasil devem ser fiscalizadas, ampliadas e articuladas às ações intersetoriais de equidade e justiça social, alinhadas aos Objetivos do Desenvolvimento Sustentável (ODS), para reduzir desigualdades e aumentar os ganhos em saúde. A contínua mobilização da sociedade civil brasileira é fundamental para a proteção e o avanço das políticas públicas, conforme preconizado pela CQCT/OMS.

## Material suplementar

Material suplementar
